# Supplementary material for: A Computational Evaluation of the Steric and Electronic Contributions in Stereoselective Olefin Polymerization with Pyridylamido-Type Catalysts
Source: Molecules. 2023 Apr 27;28(9):3768. doi: 10.3390/molecules28093768 (PMC10180424; doi:10.3390/molecules28093768)
Supplement: Supplementary file 1 [file molecules-28-03768-s001.zip › molecules-2244286-supplementary.pdf]

SUPPORTING INFORMATION

A Computational Evaluation of the Steric and Electronic Contributions in Stereoselective Olefin Polymerization with Pyridylamido-type Catalysts

Olga D'Anania [1], Claudio De Rosa [2] and Giovanni Talarico [2,*]

[1]Scuola Superiore Meridionale, Largo San Marcellino, 80138 Napoli, Italy

[2]Dipartimento di Scienze Chimiche, Università degli Studi di Napoli Federico II, 80124 Napoli, Italy

*Correspondence: talarico@unina.it

SUPPORTING INFORMATION CONTENT

Table S1: Experimental data set for propene polymerization promoted by the analyzed catalytic systems **Page S2**

Table S2: NEDA decomposition for ΔE_{Int} term **Page S3**

Table S3: DFT electronic energies with and without dispersion corrections **Page S4**

Table S4: Effect of dispersion corrections on each energetic term **Page S5**

Figure S1: propene insertion TSs for system **IIc** **Page S6**

Figure S2: propene insertion TSs for system **Ib** **Page S6**

Figure S3: propene insertion TSs for system **IIb** **Page S7**

Figure S4: steric maps for systems **Ib, IIb, IIe** and **IVa** **Page S7**

Figure S5: propene insertion TSs for system **IIe** **Page S8**

Figure S6: propene insertion TSs for system **IVa** **Page S9**

Figure S7: propene deformation with respect to the free optimized olefin **Page S9**

References **Page S10**

Table S1. Experimental data set for propene polymerization promoted by the analyzed catalytic systems [1-6].

**Systems**	M_n (kg/mol)[(a,b)]	M_w/M_n[(a,b)]	**[*mmmm*%]** [c)]	T_m (°C) [d)]	$T_{pol.}$ (°C)	**FTIR (C.I.)**[e)]
**Ia**	124 [a)]	1.05 [a)]	80	120	20	
**Ib** [f)]	-	-	-	-	-	-
**IIa**	266 [b)]	1.2 [b)]	91	133	20	-
**IIb** [f)]	-	-	-	-	-	-
**IIc**	43	1.6	-		85	0.2
**IId**	142 [b)]	1.2 [b)]	92	140	20	-
**IIe**	198	3.0	94	154	90	-
**IIIa**	18 [a)]	1.7 [a)]	-	131	70	-
**IIIb**	22 [a)]	1.7 [a)]	-	135	70	-
**IVa**	340	1.4	73	-	25	-

a) Determined using gel permeation chromatography in 1,2,4-$C_6H_3Cl_3$ at 135 °C vs. polystyrene standards.

b) Determined using gel permeation chromatography in 1,2,4-$C_6H_3Cl_3$ at 140 °C versus polyethylene standards.

c) Determined by integration of the methyl region of the ^{13}C NMR spectrum.

d) Determined via differential scanning calorimetry.

e) FTIR Crystallinity index: For blends of atactic and isotactic polypropylene (PP) with 0 – 70% isotactic PP, the IR ratio is proportional to the percentage of isotactic PP. For greater than 98% isotactic PP the ratio is greater than 0.95, for amorphous PP the ratio is 0.2.

f) The synthesis of complexes **Ib** and **IIb** has not been reported and they are used as model systems for DFT calculations.

Table S2. Complete NEDA analysis decomposition [7] for ΔE_{Int} (ΔE_{Int} = EL + CORE + CT) for 1,2 *re* and 1,2 *si* propene enantioface TSs at the preferred site for pyridylamido-Hf systems (**Ia**-**IIIb**) and at both diastereotopic sites for system **IVa**. Values are reported in kcal/mol.

	**1,2 *re* insertion**									**1,2 *si* insertion**								
**Systems**	**CT**	**ES**	**POL**	**XC-E(D3)**	**E(D3)**	**DEF (Cat.)**	**DEF (Prop.)**	**SE (Cat+Prop.)**	**ΔE_{Int}**	**CT**	**ES**	**POL**	**XC-E(D3)**	**E(D3)**	**DEF (Cat.)**	**DEF (Prop.)**	**SE (Cat+Prop.)**	**ΔE_{Int}**
**Ia**	-216.5	-45.4	-84.5	-49.7	-13.9	130.1	234.0	43.6	-45.7	-223.8	-48.9	-84.7	-51.4	-14.5	137.5	239.0	43.9	-46.9
**Ib**	-220.2	-46.3	-83.5	-49.9	-13.4	130.5	237.0	42.9	-45.8	-220.9	-46.9	-83.4	-50.3	-13.0	130.8	238.1	42.8	-45.5
**IIa**	-200.4	-46.1	-76.2	-47.4	-14.6	123.2	219.7	39.3	-41.9	-220.6	-49.7	-84.4	-50.1	-13.2	138.9	233.4	44.0	-45.7
**IIb**	-211.1	-46.3	-77.0	-48.1	-13.5	125.0	228.7	39.6	-42.2	-224.6	-49.2	-84.3	-50.6	-12.9	137.7	238.7	43.7	-45.1
**IIc**	-212.6	-46.3	-75.2	-47.4	-13.4	125.8	226.4	38.8	-43.0	-233.3	-51.0	-83.9	-51.3	-12.9	143.8	242.0	43.6	-46.4
**IId**	-203.9	-46.2	-78.3	-48.3	-14.1	124.8	225.1	40.4	-40.9	-226.3	-49.6	-86.7	-51.2	-12.8	140.2	241.6	45.1	-44.9
**IIe**	-207.7	-47.6	-77.8	-48.8	-14.7	127.4	227.2	40.1	-41.9	-220.8	-48.9	-87.7	-51.0	-14.3	138.2	239.0	45.7	-45.3
**IIIa**	-209.9	-49.1	-74.3	-48.3	-14.3	130.6	224.8	38.5	-40.5	--231.7	-50.2	-84.5	-50.4	-13.0	141.3	242.6	44.0	-45.9
**IIIb**	-212.3	-50.1	-73.8	-48.8	-14.3	133.2	225.8	38.2	-40.4	-233.6	-54.8	-79.9	-51.2	-12.5	149.6	238.9	41.9	-43.5
**IVa (Site1)**	-225.2	-86.9	-39.8	-51.8	-13.8	140.4	232.4	21.02	-44.7	-231.0	-90.0	-40.1	-53.3	-13.6	145.7	237.4	21.2	-45.0
**IVa (Site2)**	-232.3	-87.9	-58.3	-54.8	-14.2	145.3	254.5	30.7	-47.8	-226.6	-85.0	-58.2	-53.5	-13.8	140.6	249.9	30.5	46.5

EL = ES + POL + SE; CORE = XC + DEF - SE.

ES = classical electrostatic interaction; POL = polarization interaction; SE = linear response energy penalty of polarization; XC = exchange-correlation interaction; DEF = energy required to deform the wavefunctions of a fragment in the presence of all other fragments; CT = charge transfer relative to the occupied - empty orbitals interaction; XC values reported in table do not include dispersion contribute; E-D3 energy contribute for Grimme-D3 dispersion.

Table S3. DFT electronic energies (Gibbs energies) in kcal/mol, with and without dispersion corrections, for the propene stereoselectivity for the studied systems.

Systems	$\Delta E(\Delta G)_{Stereo}$[a)]	$\Delta E(\Delta G)_{Stereo}$[b)]	$\Delta E(\Delta G)_{Stereo}$[c)]	$\Delta E(\Delta G)_{Stereo}$[d)]
**Ia**	2.4 (1.4)	2.8 (1.4)	1.2(0.1)	1.6(0.5)
**Ib**	-0.6 (-0.2)	-0.5 (-0.1)	-0.6(-0.2)	-0-4(-0.2)
**IIa**	4.0 (3.1)	3.6 (2.8)	2.8(1.1)	1.7(0.8)
**IIa (OptD3)**[e)]	4.3(3.9)	4.2(3.7)	-	-
**IIb**	0.8 (0.5)	0.5 (0.2)	-0.2(-0.5)	-0.4(-0.7)
**IIc**	1.2 (1.4)	1.3 (2.0)	0.6(1.0)	0.7(1.0)
**IId**	3.8 (3.1)	3.2 (2.5)	2.3(1.6)	1.6(1.0)
**IIe**	3.8 (4.4)	3.8 (4.1)	2.1(1.9)	1.7(1.5)
**IIIa**	2.2 (2.5)	2.2 (2.5)	0.5(0.8)	0.5(0.7)
**IIIb**	4.6 (4.1)	4.6 (4.1)	2.6(2.1)	2.6(2.1)
**IVa** (site 1)	1.8 (2.4)	2.2 (2.4)	1.3(1.8)	1.7(2.2)
**IVa** (site 2)	3.1 (2.4)	3.6 (2.9)	1.9(1.2)	2.4(1.8)

[a)]DFT electronic energies (free energies) for the stereoselectivity of the studied systems including dispersion and solvent corrections (PCM model) in single-point calculations. Differences are calculated with respect to the favored propene enantioface insertion TS (1,2 *re*).

[b)]DFT electronic energies (free energies) for the stereoselectivity of the studied systems including dispersion corrections in single-point calculations. Differences are calculated with respect to the favored propene enantioface insertion TS (1,2 *re*).

[c)]DFT electronic energies (free energies) for the stereoselectivity of the studied systems including just solvent corrections (PCM model) in single-point calculations. Differences are calculated with respect to the favored propene enantioface insertion TS (1,2 *re*).

[d)]DFT electronic energies (free energies) for the stereoselectivity of the studied systems including neither solvent corrections (PCM model) nor dispersions in single-point calculations. Differences are calculated with respect to the favored propene enantioface insertion TS (1,2 *re*).

[e)]DFT electronic energies (free energies) for the stereoselectivity of system **IIa** obtained through optimization calculations including dispersion corrections. Differences are calculated with respect to the favored propene enantioface insertion TS (1,2 *re*).

Table S4. Effect of dispersion corrections inclusion on each energetic term obtained through the ASM-NEDA scheme for selected systems.

		1,2 *re* insertion					1,2 *si* insertion					1,2 (*si*-*re*) insertion[a)]				
		ΔE_{Tot}	ΔE_{Int}	ΔE_{Strain}	ΔE_{Strain} (Cat)	ΔE_{Strain} (Mon)	ΔE_{Tot}	ΔE_{Int}	ΔE_{Strain}	ΔE_{Strain} (Cat)	ΔE_{Strain} (Mon)	$\Delta\Delta E_{Tot}$	$\Delta\Delta E_{Int}$	$\Delta\Delta E_{Strain}$	$\Delta\Delta E_{Strain}$ (Cat)	$\Delta\Delta E_{Strain}$ (Mon)
**IIb**	**D3**	-1.4	-42.2	40.9	25.3	15.6	-0.9	-45.1	44.2	25.1	19.2	0.5	-2.9	3.4	-0.2	3.5
	**Without D3**	9.4	-28.7	38.1	22.4	15.7	8.9	-32.3	41.2	21.9	19.3	-0.5	-3.6	3.1	-0.5	3.6
**IId**	**D3**	-4.0	-40.9	36.9	22.2	14.7	-0.8	-44.9	44.1	24.7	19.4	3.2	-4.1	7.2	2.5	4.7
	**Without D3**	8.7	-26.8	35.5	20.7	14.8	10.3	-32.1	42.4	22.9	19.5	1.6	-5.3	6.9	2.2	4.7
**IIIb**	**D3**	-1.3	-40.4	39.1	23.0	16.1	3.1	-43.6	46.7	24.4	22.3	4.4	-3.2	7.6	1.4	6.2
	**Without D3**	12.1	-26.0	38.1	21.9	16.2	14.7	-30.9	45.6	23.2	22.4	2.6	-4.9	7.5	1.3	6.2

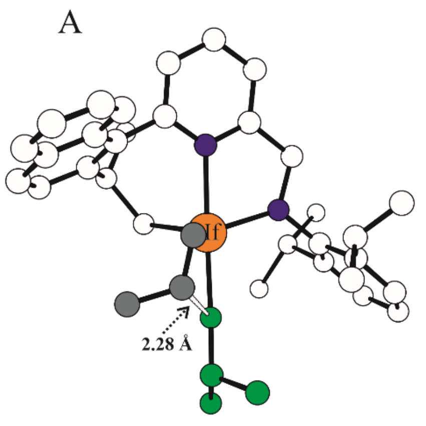

Figure S1. DFT optimized geometries for 1,2 propene insertion TS into the growing polymer chain with *re* (A) and *si* enantioface (B) for **IIc** system. Propene is shown in grey and the polymer chain (represented by ^{i}Bu group) in green. The dashed red arrows show the ligand-monomer interactions and H atoms omitted for clarity.

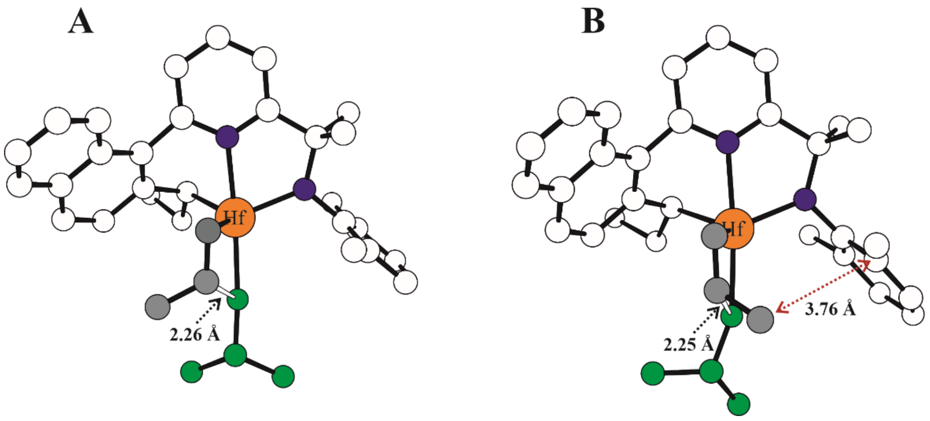

Figure S2. DFT optimized geometries for 1,2 propene insertion TS into the growing polymer chain with *re* (A) and *si* enantioface (B) for **Ib** system. Propene is shown in grey and the polymer chain (represented by ^{i}Bu group) in green. The dashed red arrows show the ligand-monomer interactions and H atoms omitted for clarity.

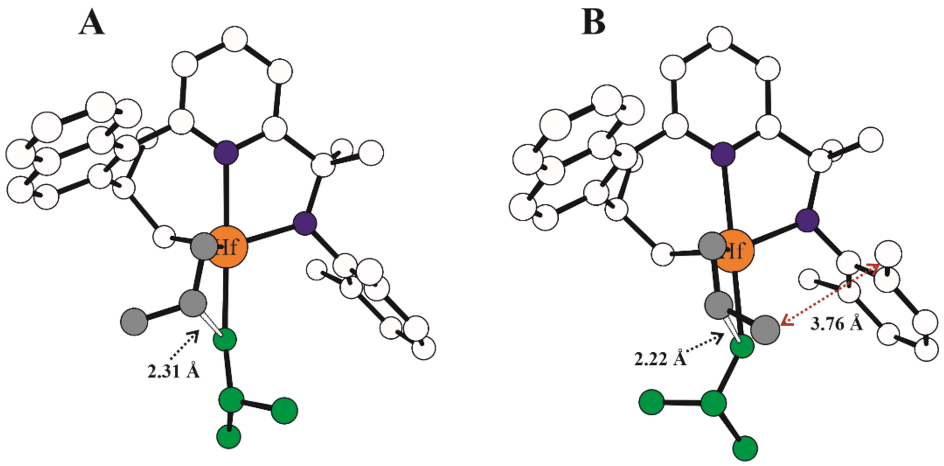

Figure S3. DFT optimized geometries for 1,2 propene insertion TS into the growing polymer chain with *re* (A) and *si* enantioface (B) **IIb** system. Propene is shown in grey and the polymer chain (represented by ^{i}Bu group) in green. The dashed red arrows show the ligand-monomer interactions and H atoms omitted for clarity.

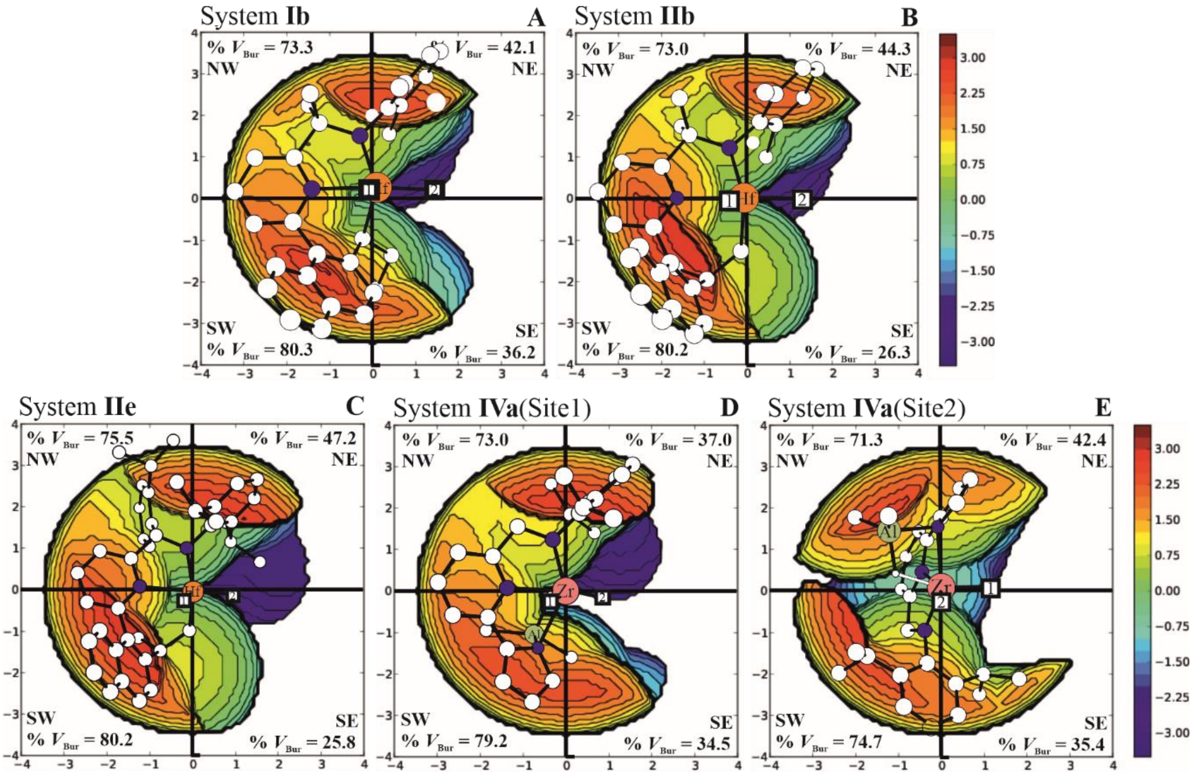

Figure S4. Steric maps of neutral mono-inserted species for systems **Ib** (A), **IIb** (B), **IIe** (C), **IVa** (Site1) (D) and **IVa** (Site2) (E) with the %V_{Bur} values obtained for each quadrant. Propene is reported in yellow. The scale, in Å, is represented on the right. The steric hindrance of the ligand framework is described through a color scale, which ranges from red to blue. The red and blue areas indicate the more- and less-hindered zones, respective.

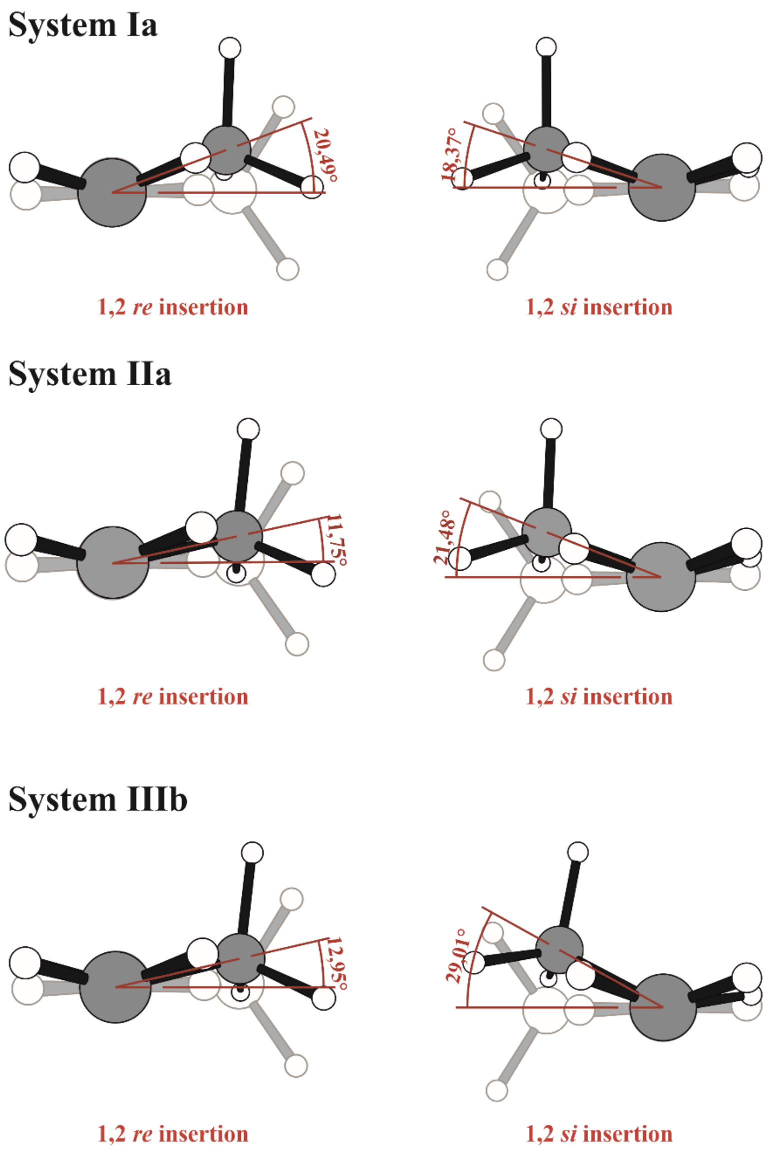

Figure S5. Representation of propene (dark grey) deformation with respect to the free optimized olefin (light grey) for 1,2 *re* and 1,2 *si* insertions with **Ia**, **IIa** and **IIIb** systems.

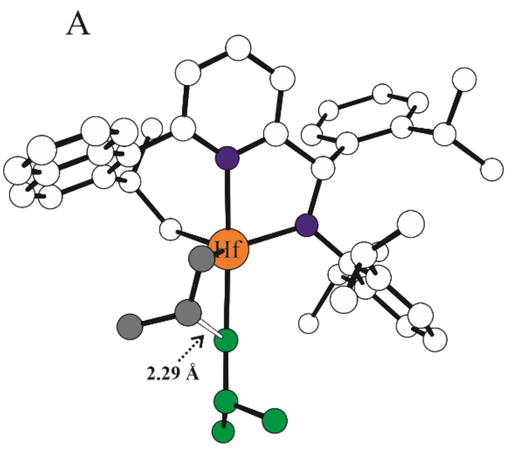

Figure S6. DFT optimized geometries for 1,2 propene insertion TS into the growing polymer chain with *re* (A) and *si* enantioface (B) for **IIe** system. Propene is shown in grey and the polymer chain (represented by ^{i}Bu group) in green. The dashed red arrows show the ligand-monomer interactions and H atoms omitted for clarity.

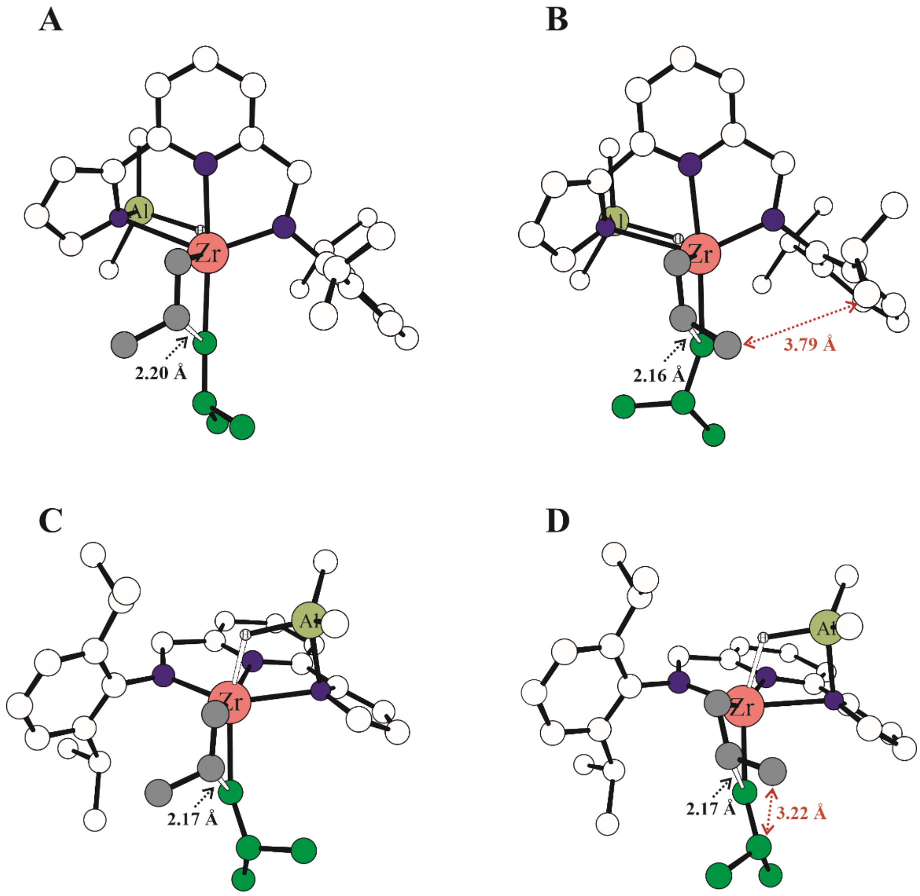

Figure S7. DFT optimized geometries for 1,2 propene insertion TS into the growing polymer chain with *re* (A,C) and *si* enantioface (B,D) for the two diastereotopic sites of system **IVa**. The stereoselectivity at site 1 (A and B) is dictated by the direct ligand-monomer interaction whereas the propene enantioface selection at site 2 (C and D) occurs following the Corradini model [8]. The kinetic preference for site 2 is below 1 kcal/mol [1]. Propene is shown in grey, polymer chain (represented by ^{i}Bu group) in green and H atoms omitted for clarity.

References

1. De Rosa, C.; Di Girolamo, R.; Muñoz-García, A. B.; Pavone, M.; Talarico, G. Breaking symmetry rules enhance the options for stereoselective propene polymerization catalysis. *Macromolecules* **2020**, 53, 2959-2964. DOI: 10.1021/acs.macromol.0c00280.
2. Domski, G. J.; Eagan, J. M.; De Rosa, C.; Di Girolamo, R.; LaPointe, A. M.; Lobkovsky, E. B.; Talarico, G.; Coates, G. W., Combined Experimental and Theoretical Approach for Living and Isoselective Propylene Polymerization ACS Catal. **2007**, 7, 6930-6937. DOI: 10.1021/acscatal.7b02107
3. Boussie, T. R.; Diamond, G. M.; Goh, C.; Hall, K. A.; LaPointe, A. M.; Leclerc, M. K.; Murphy, V.; Shoemaker, J. A. W.; Turner, H.; Rosen, R. K.; Stevens, J. C.; Alfano, F.; Busico, V.; Cipullo, R.; Talarico, G. Nonconventional Catalysts for Isotactic Propene Polymerization in Solution Developed by Using High-Throughput-Screening Technologies. *Angew. Chem., Int. Ed.* **2006**, *45*, 3278-3283. DOI: 10.1002/anie.200600240
4. Annunziata, L.; Pappalardo, D.; Tedesco, C.; Pellecchia, C. Isotactic-specific polymerization of propene by a C_s-symmetric zirconium (IV) complex bearing a dianionic tridentate [−NNN−] amidomethylpyrrolidepyridine ligand. *Macromolecules* **2009**, *42*, 5572-5578. DOI: 10.1021/ma900883t
5. Domski, G. J.; Edson, J. B.; Keresztes, I.; Lobkovsky, E. B.; Coates, G. W. Synthesis of a new olefin polymerization catalyst supported by an sp^3-C donor via insertion of a ligand-appended alkene into the Hf–C bond of a neutral pyridylamidohafnium trimethyl complex. *Chem. Commun*. **2008**, 6137-6139. DOI: 10.1039/B811384J
6. Kulyabin, P. S.; Uborsky, D. V.; Voskoboynikov, A. Z.; Canich, J. A. M.; Hagadorn, J. R. Pyridylamido hafnium complexes with a silylene bridge: synthesis and olefin polymerization. *Dalton Trans.* **2020**, *49*, 6693-6702. DOI: 10.1039/D0DT01031F.
7. Glendening, E. D. Natural Energy Decomposition Analysis: Explicit Evaluation of Electrostatic and Polarization Effects with Application to Aqueous Clusters of Alkali Metal Cations and Neutrals. *J. Am. Chem. Soc.* **1996**, *118*, 2473-2482. DOI: 10.1021/ja951834y
8. Corradini, P.; Guerra, G.; Cavallo, L. Do New Century Catalysts Unravel the Mechanism of Stereocontrol of Old Ziegler-Natta Catalysts? *Acc. Chem. Res.* **2004,** *37*, 231-241. DOI: 10.1021/ar030165n.
